# 1299. Clinical Characteristics of Native Vertebral Osteomyelitis in Patients with a History of Acupuncture

**DOI:** 10.1093/ofid/ofad500.1138

**Published:** 2023-11-27

**Authors:** Soo-youn Moon, Kyoung Ree Lim, Jun Seong Son

**Affiliations:** Kyung Hee University Hospital at Gangdong, Seoul, Seoul-t'ukpyolsi, Republic of Korea; Kyung Hee University Hospital at Gangdong, Seoul, Seoul-t'ukpyolsi, Republic of Korea; Kyung Hee University Hospital at Gangdong, Seoul, Seoul-t'ukpyolsi, Republic of Korea

## Abstract

**Background:**

Considering that vertebral osteomyelitis (VO) can occur via various routes. It can be predicted that clinical characteristics may vary depending on the way of infection or risk factors of the disease. In this study, the clinical characteristics, causative pathogens, clinical features, and prognosis of the native VO in patients with a history of acupuncture were compared with those without a history of acupuncture.

**Methods:**

This retrospective study was conducted at a university-affiliated hospital in Seoul, from May 2006 to February 2021. The patients of age 18 or older with native VO were enrolled. Data on demography, clinical presentation, treatment, causative organisms, and clinical outcomes were collected via electronic medical records.

**Results:**

A total of 100 patients with VO were reviewed, among which 34 patients had a history of acupuncture before the diagnosis of VO. The frequency of Gram-positive cocci (GPC) was significantly higher in the acupuncture group than in the non-acupuncture group (*p*=0.016). Abscess was observed more frequently in the acupuncture group than in the non-acupuncture group (*p*=0.01). Patients with abscesses underwent surgical treatment more often than those without (p=0.021). There was no difference in clinical outcomes between the two groups, including mortality, neurologic sequelae, and recurrent after one year.

**Conclusion:**

This study suggests that confirming a history of acupuncture may help predict the pathogen or clinical characteristics of the disease. If the patient has a history of acupuncture, GPC can be considered the causative organism. The presence of abscesses may lead to surgical treatment more frequently.

**Disclosures:**

**All Authors**: No reported disclosures

